# Endoscopic hand-suturing for endoscopic submucosal dissection wound closure: a didactic clinical case presentation

**DOI:** 10.1055/a-2701-5021

**Published:** 2025-10-07

**Authors:** Federico Barbaro, Tommaso Schepis, Michele Francesco Chiappetta, Rossella Maresca, Rosario Landi, Cristiano Spada

**Affiliations:** 1 18654 Digestive Endoscopy Unit, Fondazione Policlinico Universitario Agostino Gemelli IRCCS, Rome, Italy; 2 Centre for Endoscopic Research Therapeutics and Training (CERTT), Università Cattolica Del Sacro Cuore di Roma, Rome, Italy

Endoscopic submucosal dissection (ESD) allows the en-bloc resection of large gastrointestinal lesions, enabling precise pathological evaluation and oncological radicality 1 2 . Post-ESD wound closure can reduce delayed adverse events (AEs) 3 , but standard devices struggle with large GI-defect closures. The introduction of new suturing allows for effective solutions. To date, limited studies evaluated the feasibility of hand-suturing for post-ESD wound closure 4 .

We report the case of a 68-year-old male diagnosed with a 40-mm nongranular laterally spreading tumor located in the distal rectum (Paris Classification 0-IIa + IIc, IV with focal Vi Kudo Class, 2A and focal 2B JNET Class) ( Fig. 1 ). ESD was performed using the GIF-H190 scope and DualKnifeJ electrosurgical knife (Olympus Medical Systems). The rich vascular pattern, intraprocedural bleedings, and patients’ need for early anticoagulation treatment reintroduction (atrial fibrillation as comorbidity) significantly increased the risk of delayed bleeding. An endoscopic needle-holder (FG-260, SutuArt; Olympus Medical Systems) and a V-Loc 180 suture, size 3–0 (Medtronic), were used for complete post-ESD wound closure ( Video 1 ).

**Fig. 1:**
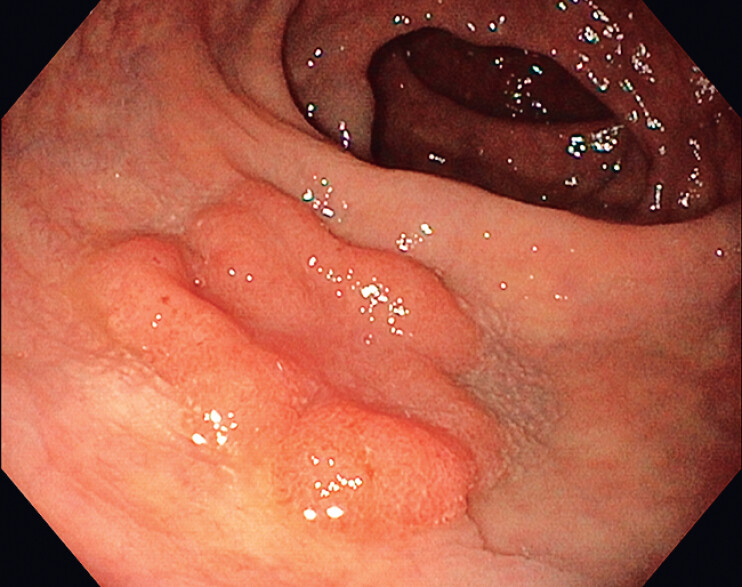
A 40-mm large nongranular laterally spreading tumor of the distal rectum (Paris Classification 0-IIa + IIc) with pit pattern type IV, focally Vi according to Kudo Classification, vascular pattern type 2A + 2B in the depressed area according to JNET classification.

Post-ESD wound closure, in a patient at high risk for delayed bleeding, using an endoscopic needle-holder (FG-260, SutuArt; Olympus Medical Systems) and a V-Loc 180 suture, size 3–0 (Medtronic).Video 1

Suturing followed these steps:

1. The needle was introduced with the tip grasped by the needle holder inside a distal attachment.
2. Once in the target area, the needle was grasped by the needle-holder with the tip pointed to the left; the needle should be grasped at 1/3–1/2 of the needle length away from the end and always perpendicular to the needle holder.
3. The tip was pointed at the proximal edge of the wound.
4. The suture was pulled after each stitch placement to tight the suture.
5. A continuous suture was performed until the wound was completely closed
6. A final stitch was placed in the direction opposite to the stitching direction.
7. Suture cut (loop cutter FS-410; Olympus Medical Systems).

The procedure was completed successfully ( Fig. 2 , Fig. 3 ), follow-up was uneventful, and no AEs were recorded.

**Fig. 2:**
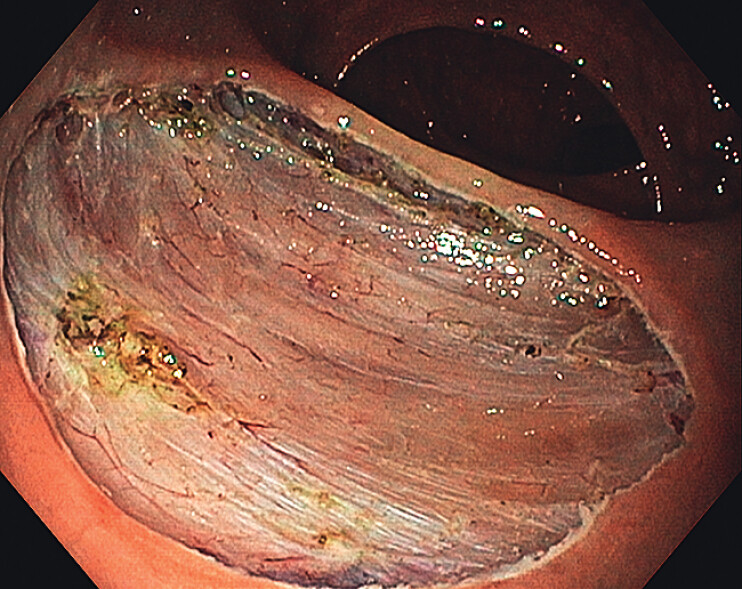
Post-ESD wound after complete en-bloc resection.

**Fig. 3:**
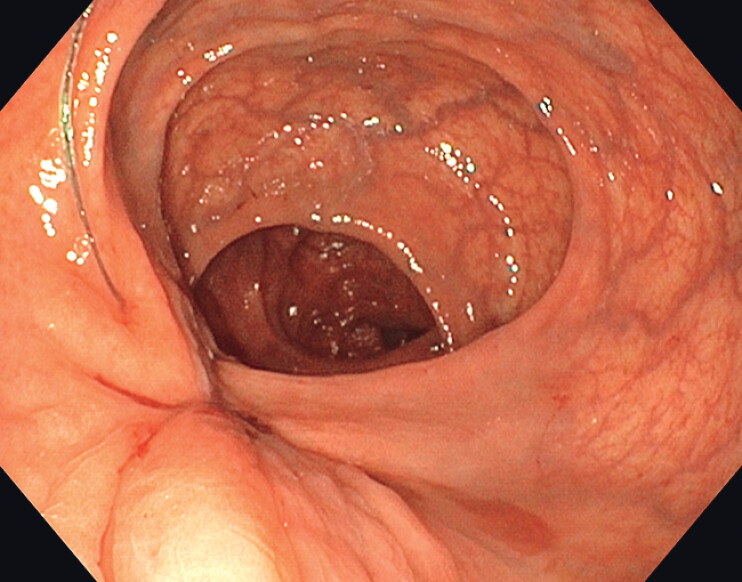
Complete wound closure with SutuArt.

Endoscopy_UCTN_Code_TTT_1AQ_2AK

## Notes

Endoscopy E-Videos https://eref.thieme.de/e-videos
*E-Videos* is an open access online section of the journal *Endoscopy* , reporting on interesting cases and new techniques in gastroenterological endoscopy. All papers include a high-quality video and are published with a Creative Commons CC-BY license. Endoscopy E-Videos qualify for HINARI discounts and waivers and eligibility is automatically checked during the submission process. We grant 100% waivers to articles whose corresponding authors are based in Group A countries and 50% waivers to those who are based in Group B countries as classified by Research4Life (see: https://www.research4life.org/access/eligibility/ ). This section has its own submission website at https://mc.manuscriptcentral.com/e-videos .
